# Comparison of the overall fit of three-unit posterior fixed dental prostheses fabricated with laser sintering and conventional casting methods

**DOI:** 10.1007/s00784-025-06221-8

**Published:** 2025-02-24

**Authors:** Gürel Pekkan, Kubra Degirmenci, Süleyman Hakan Tuna, Canan Hekimoğlu, Serkan Saridag

**Affiliations:** 1 Department of Prosthodontics, Faculty of Dentistry, Tekirdag Namik Kemal University, Tekirdag, Turkey; 2 Department of Prosthodontics, Faculty of Dentistry, Bolu Abant Izzet Baysal University, Bolu, Turkey; 3 Department of Prosthodontics, Faculty of Dentistry, Dokuz Eylül University, Izmir, Turkey; 4 Department of Prosthodontics, Faculty of Dentistry, Hacettepe University, Ankara, Turkey; 5 Department of Prosthodontics, Faculty of Dentistry, Istanbul Okan University, Istanbul, Turkey

**Keywords:** Laser sintering, Co-cr, Fixed dental prostheses, 3D-printing, CAD/CAM, Discrepancy

## Abstract

**Objectives:**

The aim of this study was to investigate the marginal, internal, and occlusal discrepancies of three-unit posterior cobalt-chromium (Co-Cr) fixed dental prostheses (FDPs) produced using five different fabrication techniques.

**Materials and methods:**

Segmental maxillary models were prepared from polyamide material using a laser sintering method. The maxillary first premolar and first molar teeth were prepared to receive posterior FDPs. Direct metal laser sintering (DMLS) and selective laser melting (SLM) were used as two metal laser Co-Cr framework production systems. FDP specimen patterns were prepared by manual wax carving (Cast), 3D-printed polymer (3DP), and CAD/CAM wax and cast using the lost-wax technique as conventional methods. In total, 100 Co-Cr metal framework specimens were prepared for posterior FDPs (*n* = 20). The silicone replica technique was used to measure marginal, internal, and occlusal discrepancies of all frameworks. A stereomicroscope was employed to detect discrepancies at 100× magnification. The data were analyzed using two-way ANOVA (α = 0.05) and post hoc Bonferroni adjustment (α = 0.005) for pairwise comparisons.

**Results:**

There were no significant differences between the occlusal discrepancy values of premolar abutments of FDPs when compared with different fabrication methods (*P* > 0.05). The highest marginal discrepancy value was detected as 116.22 μm for molar abutment when the Cast method was used (*P* < 0.05). The highest occlusal discrepancy values were detected as 135.60 μm and 141.49 μm for molar abutments of posterior FDPs when the 3DP and Cast methods were used. The lowest marginal discrepancy value was detected as 38.94 μm for molar abutments when the DMLS method was used (*P* < 0.05).

**Conclusions:**

The DMLS method was more successful than other fabrication methods when fit values of abutment teeth for posterior frameworks were compared.

**Clinical relevance:**

The morphology of the abutment teeth and the fabrication techniques of FDPs migth affect the discrepancy values of FDPs planned. It was seen that the discrepancy values were lowest with the DMLS and SLM methods. Considering the results of this in-vitro study, DMLS and SLM techniques may be more appropriate option than the 3DP method, which starts with digital design and ends conventionally casting technique for posterior three-unit FDPs.

## Introduction

Marginal and internal adaptation between the restoration and abutment tooth is crucial for the biomechanical strength of the restoration–tooth complex. Successful marginal and internal adaptation enhance the fracture resistance of the restoration [1, 2]. Marginal adaptation can be affected by tooth preparation, and it is important for the structural strength of fixed dental prostheses (FDPs) [3]. Similarly, the internal fit is important for achieving the clinical success of FDPs [4]. Stress concentrations may be reduced with improvements in the internal fit, which may increase the strength of dentures [5]. The internal fit is evaluated by measuring the vertical difference between the internal surface of the framework and the axial wall of the prepared abutment [6]. The marginal adaptation of a restoration can be measured by evaluating the fit between the margins of the restoration and the prepared abutment teeth [7–9]. Marginal discrepancies between the abutment teeth and restoration behave as a reservoir for microorganisms, and eventually, caries and periodontal problems can be seen on abutment teeth [9]. Although it was reported that marginal discrepancy values (MDVs) less than 120 μm should be considered clinically acceptable, there is no consensus on the maximum clinically acceptable MDV [2, 10].

Recently, the use of cobalt-chromium (Co-Cr) alloys to produce the metal framework of FDPs has increased [11]. Co-Cr alloys have ideal mechanical properties, and the material is inexpensive when compared with other precious alloys [12, 13]. Co-Cr frameworks can be fabricated with both conventional casting (lost-wax) and laser sintering methods. The fabrication technique may affect the marginal and internal adaptation of the metal framework [14].

To produce a metal framework with the conventional casting method, a wax pattern is prepared on the abutment teeth. Thereafter, investing, wax elimination, and casting are achieved, respectively [15]. However, the conventional method is time-consuming and requires a technician’s skills to achieve an eligible wax pattern [16]. Additionally, wax material has some disadvantages, such as thermal sensitivity, fragility, and a high coefficient of thermal expansion [4, 17]. Recently, computer-aided design and computer-aided manufacturing (CAD-CAM) systems have been used in combination with conventional casting methods [13, 17]. 3D-printed polymer (3DP) or CAD-CAM wax blocks may be preferred over manual carving of the wax pattern. After the design in the CAD program, wax patterns are achieved with CAM systems by subtractive manufacturing from wax blocks, or polymer patterns are produced with 3D-printing systems, and the wax or polymer patterns are cast with the conventional casting method [16].

After the metal framework is designed in the CAD program, it can be produced directly with selective laser melting (SLM) or selective laser sintering (SLS) [18, 19]. SLM and SLS systems are additive manufacturing methods, and a high-energy carbon-dioxide laser beam is used to fuse or sinter Co-Cr metal powder particles in layers until the designed framework is obtained [20]. Furthermore, direct metal laser sintering (DMLS) was introduced as an SLS method. One or multiple laser beams are used to construct a metal framework from metal powders by sintering approximately 20- to 60-µm-thick layers at each shooting [21]. The general advantages of laser sintering systems are saving time and eliminating technician-dependent errors and material-dependent limitations in wax carving, investing, and casting procedures.

On the other hand, the marginal and internal fit of the restorations may be different depending on the abutment teeth, even if the fabrication method was the same [8, 22]. Limited studies have comparatively evaluated three-unit Co-Cr dental restoration substructure fabrication methods for different abutment teeth [23]. Although the preparation principles are general, morphological differences still exist after the preparations, and these may impact the fit of FDPs [22]. There are no definite conclusions regarding the effect of different fabrication methods on the marginal and internal fit in planning FDPs in posterior teeth [2, 15, 17, 20]. Previous studies that comparatively evaluated the effect of various fabrication methods on the adaptation of FDPs prepared in different posterior abutment teeth are not sufficient to determine a consensus [7, 8, 10].

The purpose of this study was to compare different fabrication methods for posterior FDPs and provide clinical insights into the effect of fabrication methods on the fit of FDPs prepared for different types of abutment teeth. The first null hypothesis of the study was that the Co-Cr metal framework fabrication method would have no effect on the marginal, occlusal, and internal fit of the three-unit posterior FDP framework. The second null hypothesis was that there would be no differences between the marginal, occlusal, and internal fit of three-unit posterior FDPs created with the same fabrication method.

## Methods

Sample size and prior power analysis were achieved with G*Power v3.1 [24] for “Means – Many groups ANOVA: main effects and interactions.” A medium effect size f of 0.25 with alpha 0.05, power 0.80, numerator df 4 (five manufacturing methods and molar-premolar abutment types), and number of groups 10 resulted in the required number of specimens, 196. Therefore, 200 abutment teeth specimens and 20 FPD framework specimens were prepared per fabrication group.

**Fig. 1:**
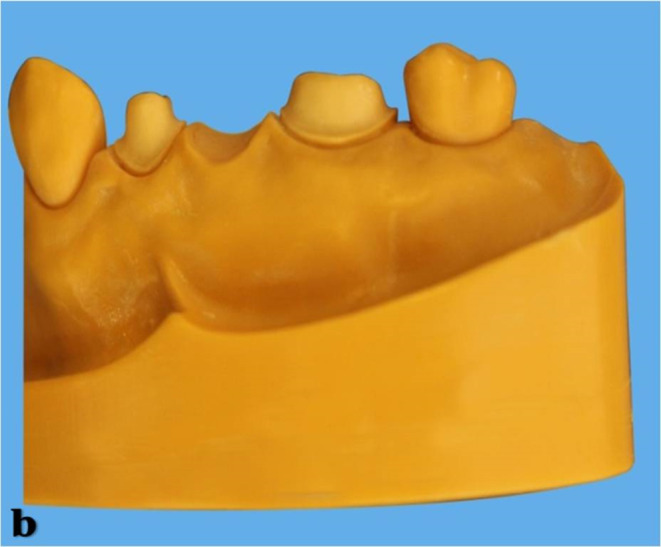
The maxillary segmented master model with prepared first premolar and first molar teeth

With a laser plastic sintering machine (Formiga P 110, EOS GmbH, Krailling, Germany), a maxillary master model was prepared from fine polyamide powder (Polyamide 12, PA 2105; EOS GmbH). Three-unit FDPs were prepared for posterior regions on the segmented master models. For posterior FDPs, right first premolar and first molar teeth were prepared (Fig. 1).

Preparations of the abutment teeth were made according to the principles of general tooth preparation: 1.5 to 2 mm on the occlusal surfaces, 1 to 1.5 mm on the axial surfaces, and a convergence angle of approximately 6 degrees [25, 26] with chamfer finish lines. Thereafter, prepared teeth in the master models were evaluated with a parallelometer (Dental Surveyor; Unident Instrument Pvt., Ltd., New Delhi, India) to prevent undercut formation. After final adjustments were made, digital or conventional impressions were made from the master model according to the fabrication method used for each group.

**Fig. 2:**
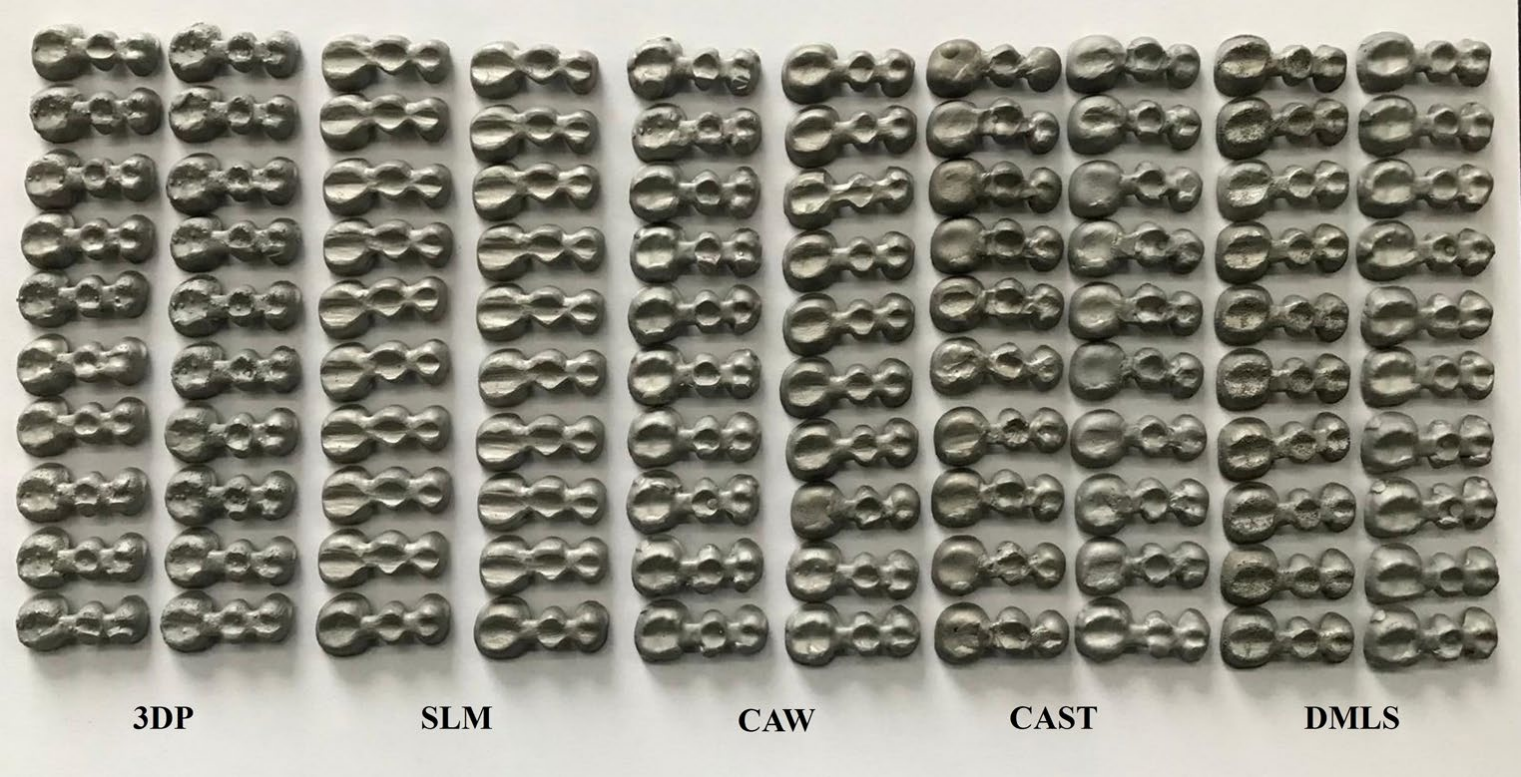
Specimens of five different groups of maxillary 3-unit posterior FDP frameworks

Five different fabrication methods were utilized to prepare a total of 100 Co-Cr three-unit FDP metal framework specimens (*n* = 20) (Fig. 2).

### DMLS

Three-dimensional models were made by scanning the master model with the prepared teeth with a desktop scanner (DentalWings 7 Series; DentalWings, Montreal, Canada). The design program (DWOS crown and bridge CAD Software, DentalWings) was used to virtually design the metal frameworks. The copings of Co-Cr FDP frameworks were set as 0.5 mm in thickness. The virtual design was adjusted to obtain a 70-µm cement space on the entire inner surface 0.5 mm from the margin levels. The connector areas of the frameworks were set to 7 mm^2^ for FDP specimens. A total of 20 Co-Cr three-unit FPD frameworks were produced by the DMLS machine (EOS M 270; EOS GmbH), using its recommended powder (SP2: EOS SP2; Turku, Finland), according to the manufacturer’s instructions.

### SLM

The same design procedure as in the DMLS group was followed, and a total of 20 Co-Cr three-unit FDP frameworks were produced by the SLM machine (ConceptLaser Mlab, ConceptLaser GmbH, Lichtenfels, Germany) according to the manufacturer’s instructions using its recommended powder (Remanium^®^ star; Dentaurum, Ispringen, Germany).

### Manual wax carving (cast)

Impressions of the master model were made with vinyl polysiloxane (VPS) (Elite HD + Putty soft; Zhermack SpA, Badia Polesine, Italy), and plaster (Hera Moldastone CN; Heraeus Kulzer GmbH, Hanau, Germany) models were obtained. Thereafter, removable die models were prepared with the Pindex system (ED-Laser; Dentalfarm Srl, Turin, Italy). Die spacers were utilized to standardize the cement space (Durolan; DFS Diamon, Riedenburg, Germany). A total of 70 μm of cement space was created by applying four layers of 15-µm-thick (Durolangold; DFS Diamon) die spacer and one layer of 10-µm-thick die spacer (Durolanblue; DFS Diamon) to abutment teeth, except 0.5-mm marginal areas. Wax patterns were prepared using modeling wax (GEO Dip gelb; Renfert GmbH, Hilzingen, Germany) melted in a melting unit (Hotty; Renfert GmbH). To standardize the dimensions of the pontics and connector parts of the cast FDP specimens, the pontics and connector parts of wax FDP patterns were milled from wax blocks (Alliance Wax Disc; Almadent, Izmir, Turkiye) using the standard CAD design and were connected to wax copings using the electric waxing unit (Hot 2 touch; Dentalfarm, Turin, Italy). After spruing, specimens were invested with an investment material (Castorit-Super C; Dentaurum, Ispringen, Germany). After the preheating and wax-elimination process at 950 °C, Co-Cr ingots (Kera C; Eisenbacher Dentalwaren ED GmbH, Main, Germany) were melted at approximately 1400 °C in an induction casting machine (INF 2010; Mikrotek Dental, Ankara, Turkiye) using a ceramic crucible (Bego Fornax; Bego, Bremen, Germany). After divestments, the specimens were examined under a 5× laboratory microscope. Specimens with unacceptable casting defects were excluded from the study, and new specimens were fabricated. A total of 20 Co-Cr FDP frameworks were prepared with the conventional casting method.

### CAD/CAM (3DP)

In this group, the specimens were prepared according to CAD designs using translucent polymeric material (Fusia RF080; DWS Srl, Thiene, Italy) utilizing a laser stereolithography machine (Digitalwax 028D; DWS Srl). The specimens were stored in a light-resistant box for 48 h and tried on the abutment teeth before casting. The polymeric FDP patterns were sprued, invested, and cast from a Co-Cr alloy (Kera C; Eisenbacher Dentalwaren ED GmbH) according to the conventional casting procedures. A total of 20 Co-Cr FDP frameworks were prepared.

### CAD/CAM Wax (CAW)

The CAW specimens were milled from CAD/CAM wax blocks (Alliance Wax; Almadent), and the specimens were prepared according to the conventional casting method described for the Cast and 3DP groups. A total of 20 Co-Cr FDP frameworks were fabricated.

**Fig. 3:**
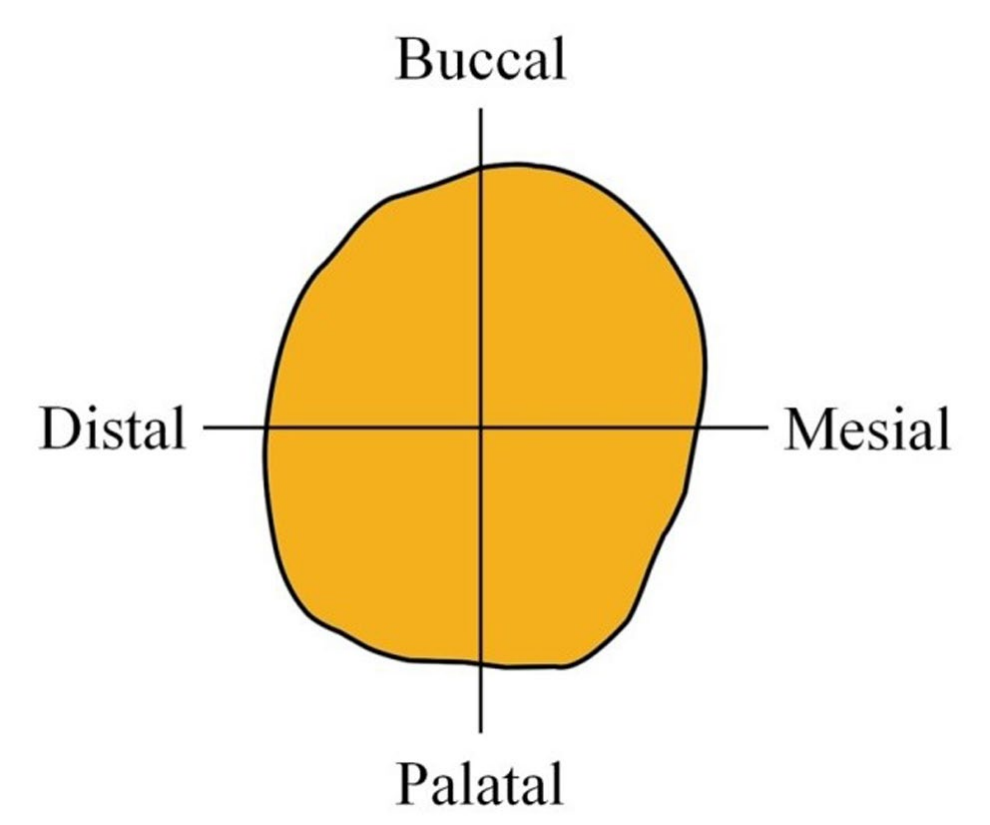
Drawing of the cutting line of the abutment teeth replicas. Silicone replicas sectioned in buccopalatal and mesiodistal directions before measurements

**Fig. 4:**
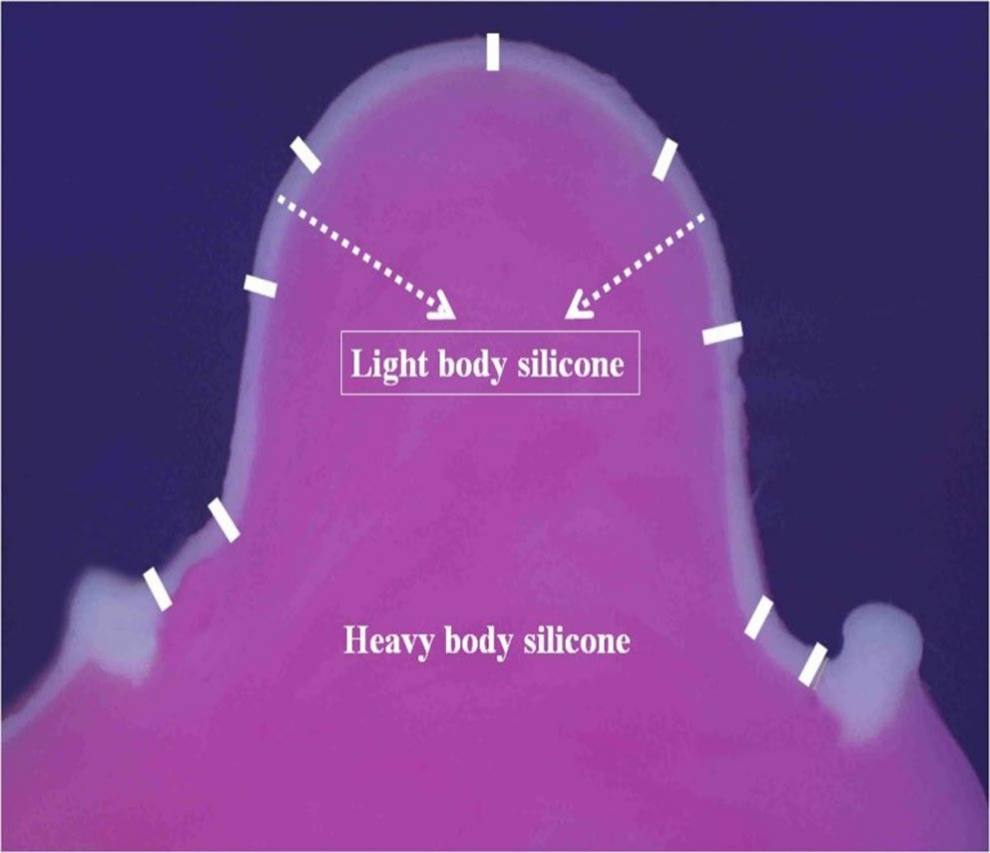
Silicone replica evaluation of a specimen under stereomicroscopy

The marginal and internal fit of the frameworks were examined with the silicone replica technique (SRT). VPS materials of different colors and viscosities were used to detect discrepancies between the three-unit FDP metal frameworks and prepared abutment teeth. VPS material (Fit Checker Advanced White; GC Corp., Tokyo, Japan) was applied to the copings of FDP frameworks. FDP frameworks were seated onto abutment teeth with finger pressure. To apply an equal load during the setting of silicone material, a vertical pressure of 40 N was applied onto the pontics of the specimens with a digital dynamometer device (Digital Force Gauge SH-500, Geratech Ltd., Hong Kong, Taiwan) for one minute. After the specimens were removed, the inside of the specimens was filled up with light-body VPS impression material (Betasil Light Vario, Müller-Omicron GmbH&Co., KG., Lindlar, Germany). Silicone indexes were cut by a razor in both the mesiodistal and buccolingual directions (Fig. 3). Marginal and internal discrepancy measurements were made under a stereomicroscope (AZ100M motorized multipurpose zoom microscope; Nikon Corp., Kanagawa, Japan) at 100× magnification (Fig. 4).

**Fig. 5:**
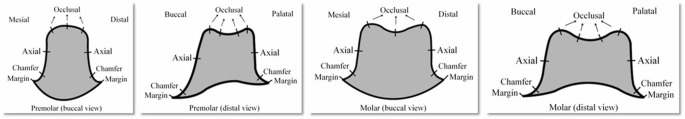
Measured regions of teeth in 3-unit posterior FDP frameworks

Nine measurement points can be seen from the buccal view in the mesiodistal direction measured for each abutment tooth (Fig. 5). Furthermore, 10 measurement points can be seen from the distal view in the bucco-palatinal direction measured for each abutment tooth (Fig. 5). A total of 19 measurement points (seven occlusal points, four axial points, four chamfer points, and four margin points) were evaluated for each abutment tooth, and a total of 38 measurement points were evaluated for each FPD framework specimen [27]. At each measurement point, three measurements were made, and the mean discrepancy value was recorded [28]. The mean marginal (four margin points), internal (four axial points and four chamfer points) and occlusal (seven occlusal points) discrepancy values (ODVs) for each abutment tooth were obtained by calculating the mean values of all measurement points.

Statistical analysis was done with the SPSS program (Version 22). Considering the distribution of the variables, skewness, and kurtosis values, the variables revealed a normal distribution, as the values were between + 1.5 and − 1.5. The effect of abutment type and fabrication method was evaluated with two-way ANOVA (α = 0.05). Pairwise comparisons of groups were evaluated with post hoc Bonferroni adjustment (*P*_adj_. = 0.05/10, α = 0.005).

## Results

**Fig. 6:**
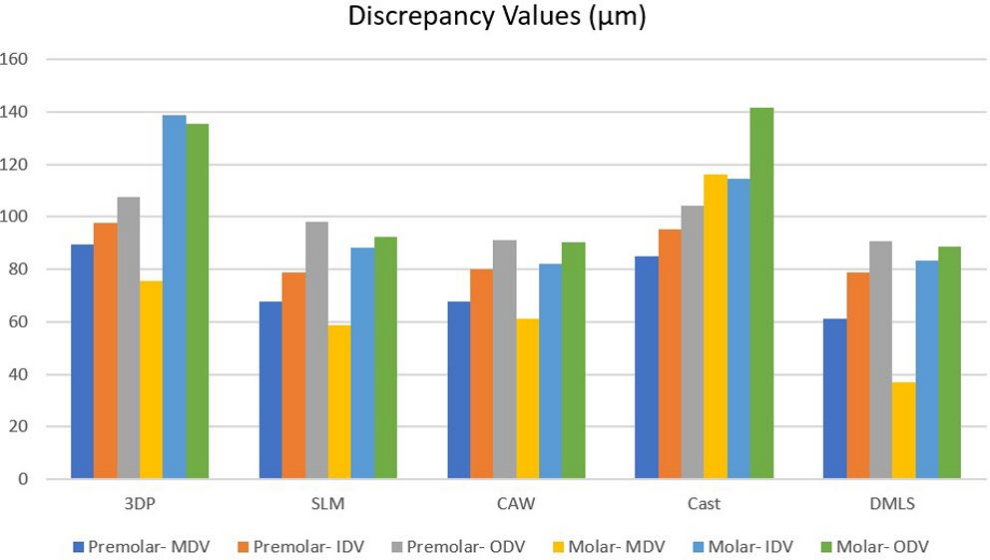
Mean discrepancy values of abutment teeth according to fabrication methods. MDV- Marginal Discrepancy Value, IDV- Internal Discrepancy Value, ODV- Occlusal Discrepancy Value

There were significant differences between the fabricating methods and the abutment types in the measurement regions for the MDV (*P* < 0.001), internal discrepancy value (IDV) (*P* < 0.001), and ODV (*P* < 0.001) (Table 1). The discrepancy results of FDPs for each abutment tooth according to the fabrication methods are graphically shown in Fig. 6.

**Table 1:** Two-way ANOVA of comparison of fabrication methods in each abutment type (marginal discrepancy Values-MDV, Internal discrepancy Values-IDV, Occlusal discrepancy Values-ODV)

Source	Sum of Squares	df	Mean Square	F	p	Partial Eta Squared
**Marginal Discrepancy Values-MDV**						
Fabrication Method	63471.707	4	15867.927	55.483	*< 0.001*	0.539
Abutment Type	1054.874	1	1054.874	3.688	0.056	0.019
Fabrication Method X Abutment Type	17773.717	4	4443.429	15.537	*< 0.001*	0.246
**Internal Discrepancy Values-IDV**						
Fabrication Method	45715.807	4	11428.952	27.561	*< 0.001*	0.367
Abutment Type	11346.205	1	11346.205	27.361	*< 0.001*	0.126
Fabrication Method X Abutment Type	9879.134	4	2469.784	5.956	*< 0.001*	0.111
**Occlusal Discrepancy Values-ODV**						
Fabrication Method	45138.795	4	11284.699	21.006	*< 0.001*	0.307
Abutment Type	6454.979	1	6454.979	12.016	*0.001*	0.059
Fabrication Method X Abutment Type	15645.952	4	3911.488	7.281	*< 0.001*	0.133

Numbers in italics indicate significant *P* values (*P* < 0.05)

Table 2 indicates vertically the comparison of measurements obtained in the marginal, internal, and occlusal regions between abutment teeth for the same fabrication method. In addition, the comparison of measurements on the same abutment tooth between different fabrication methods are horizontally shown in Table 2.

**Table 2:** Marginal, internal and, Occlusal discrepancy values of fabrication methods at different abutment types (means and standard deviations (SDs) in µm)

Measurement Region	Abutment Type	Fabrication Methods				
		3DP (Mean ± SD)	SLM (Mean ± SD)	CAW (Mean ± SD)	Cast (Mean ± SD)	DMLS (Mean ± SD)
(Marginal Discrepancy Values-MDV)	Premolar	89.52 ± 27.07^a, X^	67.91 ± 9.36^b, X^	67.78 ± 13.60^b, X^	85.14 ± 23.26^a, X^	61.33 ± 8.89^b, X^
	Molar	75.52 ± 19.42^a, X^	58.82 ± 8.31^b, X^	61.20 ± 9.74^b, X^	116.22 ± 25.47^c, Y^	36.94 ± 6.54^d, Y^
(Internal Discrepancy Values-IDV)	Premolar	97.88 ± 24.53^a, X^	78.97 ± 9.38^b, X^	80.07 ± 8.90^b, X^	95.30 ± 17.25^a, X^	78.98 ± 13.13^b, X^
	Molar	138.53 ± 29.01^a, Y^	88.05 ± 7.96^b, Y^	82.13 ± 12.81^b, X^	114.36 ± 10.15^a, Y^	83.44 ± 13.18^b, X^
(Occlusal Discrepancy Values-ODV)	Premolar	107.58 ± 28.87^a, X^	98.03 ± 16.30^a, X^	91.05 ± 18.75^a, X^	104.25 ± 25.98^a, X^	90.56 ± 11.94^a, X^
	Molar	135.60 ± 30.95^a, Y^	92.19 ± 13.05^b, X^	90.40 ± 19.35^b, X^	141.49 ± 23.35^a, Y^	88.61 ± 18.30^b, X^

The same lowercase letters (a, b, c, d) indicate that there is no significant difference in horizontal comparisons between fabrication methods (*P*_*adj*_.>0.005)

The same capital letters (X, Y) show that there is no significant difference between them in vertical comparisons between abutment teeth (*P* > 0.05)

For both the Cast and DMLS methods, there were significant differences between the MDVs of premolar and molar abutments (*P* < 0.05). When the fabrication methods were compared for molar abutment, the largest MDVs were determined for the Cast method (*P*_adj_. < 0.005), and the smallest MDVs were determined for the DMLS method (*P*_adj._< 0.005).

When IDVs were examined, significant differences were seen between premolar and molar abutments for the 3DP, SLM, and Cast fabrication methods (*P* < 0.05). There was no significant difference between the 3DP and Cast fabrication methods for the IDVs of the same abutment tooth (*P*_adj_. > 0.005). Similarly, there was no significant difference between the IDVs of the SLM, CAW, and DMLS fabrication methods for the same abutment (*P*_adj_. > 0.005).

For the 3DP and Cast fabrication methods, there were significant differences between the ODVs of premolar and molar abutments (*P* < 0.05). However, for the ODVs of premolar abutments, there were no significant differences between the fabrication methods (*P*_adj_. > 0.005). There were no significant differences between the ODVs of molar abutments when the 3DP and Cast fabrication methods (*P*_adj_. > 0.005) were used. Similarly, significant differences were not defined between the ODVs of molar abutments when the SLM, CAW, and DMLS fabrication methods were used (*P*_adj_. > 0.005).

## Discussion

This study was conducted to compare the fit of three-unit posterior FDP frameworks fabricated with different techniques. Although there were no significant differences between the fabrication methods in terms of the ODVs of FDPs for premolar abutment teeth, significant differences were detected between the fabrication methods for molar abutment teeth. There were no significant differences between the discrepancy values of different abutments when certain fabrication methods were used, but there were significant differences between the different abutment teeth for the same fabrication method according to the measured region, such as marginal, internal, and occlusal. Therefore, both the first and second null hypotheses of the study were partially accepted.

The insignificant difference in ODVs between fabrication methods for premolar abutments may be explained by the difference between the surface area of the abutment teeth. The premolar abutment has less angular morphology than the molar abutment, and the crown part of the premolar shows a more rounded form than the molar [29]. Angular areas increase the probability of framework misfit due to the technical limitations of fabrication methods [30]. The differences between the fabrication methods should be considered when preparing and working on more angular tooth surfaces. Accordingly, the highest ODVs in fabrication methods were obtained for molar abutments because the teeth inherently have a series of angles and curves on the occlusal surface [29]. Therefore, chamfer finish line prefered as the finish line because, it has been reported that chamfer finish line presented less discrepancy values and more accuracy for impressions in previous studies [31–33].

The discrepancy values of laser groups were lower than the values of 3DP, CAW, and Cast groups. This might be related to the possibility of the formation of an oxide layer in the casting process and gas porosity on irregular surfaces [34]. Furthermore, the effect of the interaction between the investment material and Co-Cr could be a reason for the higher discrepancy values [12]. Other parameters that might affect the fit of the FDPs before or during casting are the technician’s skills, contraction of wax material, and shrinkage of polymeric 3DP material [17, 30, 34].

On the other hand, the diameter of the milling bur used in the CAD/CAM system may affect the accuracy of restorations [35]. The milling bur may not sufficiently prepare sharp internal angles because of the size discrepancies between the preparation edges and the bur [36, 37]. Additionally, horizontal planes (x, y) and vertical planes (z) may affect the discrepancy values in CAM systems. Particularly errors in the z-plane may contribute to the shrinkage of the material [38, 39]. In line with this information, in this study, the IDVs and ODVs were higher than the MDVs of the 3DP and CAW groups that the CAD/CAM wax blocks were used.

Consistent with previous studies, the ODVs were higher than the MDVs in this study, and the mean ODVs measured in the posterior teeth ranged between 88.61 μm and 141.49 μm and were lower than those of the previous studies [17, 40]. Whereas flat occlusal surfaces were prepared in other studies [15, 17, 23], in the present study, the occlusal side of the premolar and molar teeth were prepared according to tooth preparation principles, and the teeth morphologies and frameworks were not cemented before discrepancy measurements were performed. Additionally, when the marginal, internal, and occlusal discrepancies between the abutment teeth and FDPs were evaluated, a general increase in the discrepancy values from the marginal to the occlusal regions was observed. This difference might be because the more detailed the occlusal or internal structure is, the more complete fit is prevented due to the increase in surface area [17]. The MDVs of the FDP substructures are affected by the prepared tooth morphology and cement thickness [2, 41]. In the present study, the aim was to standardize the cement space. Considering previous studies, the cement space prepared for all abutment teeth was set to be 70 μm [20, 42]. Thus, the narrowing in the axial regions could be hindered, and the passive fit of the FDPs to the abutment teeth was ensured [43].

Moreover, in this study, there were differences between wax pattern preparation methods. This difference could be a reason for the inconsistent discrepancy values of the substructures obtained in the Cast, CAW, and 3DP groups, although the same alloy, Co-Cr, was cast [17]. Although the lost-wax technique was used for each group, some steps, such as scanning and designing software conditions, might be responsible for discrepancy differences between the Cast, CAW, and 3DP groups [44]. Moreover, the polymerization shrinkage of 3DP polymeric material until the casting time and the light sensitivity may have affected the fit values of the 3DP group.

Marginal accuracy is effective in the structural longevity of the restoration cement. Considering the work of the researchers McLean and von Fraunhofer [10], the MDVs in all fabrication technique groups were below the clinically acceptable limit (< 120 μm). The results of this study were not in line with some of the previous studies [17, 19]. In this study, the discrepancy values for the SLS group were not lower than those of the conventional casting methods. This might be explained by the morphology of the abutment and different parameters of laser sintering systems. Örtorp et al. [22]. reported the mean internal gap as 144 ± 67 μm for the conventional Cast group and 151 ± 58 for the DMLS group. In this study, the mean IDVs were 114.36 ± 10.15 for the Cast group and 83.44 ± 13.18 for the DMLS group. This difference might be due to the measurement points and the evaluation methods used.

Conventional fabrication methods showed higher discrepancy values than the other groups for all abutments. Modeling was done manually in the Cast group and the coping margins were manually adjusted, and the distortions or ununiform thickness may have affected the adaptation of the wax specimens. Although the substructure was obtained with the conventional cast method in the Cast, CAW, and 3DP groups, the difference in the pattern preparation methods and material-dependent variations might be considered reasons for the differences between the groups [30]. The DMLS and SLM groups showed better and more consistent fit values than those of the other methods, which is in accordance with previous studies [22, 42]. However, some studies concluded that conventional cast methods showed better results than the DMLS system [21, 42]. Abutment morphology, laser beam, laser intensity, and the material used for the replica technique may have led to these results [45].

In this study, SRT was selected for discrepancy measurements because it is a convenient and inexpensive method and has been performed in previous studies [7, 46]. The SRT method is nondestructive, and its reliability is high [46]. In a previous study, micro-computed tomography and the SRT method were comparatively evaluated [47]. It was reported that the values in all the regions measured with micro-computed tomography were not significantly different from those measured using SRT [46]. However, the material used in SRT may impact the measured discrepancy values. The rupture strength of the material, the ability to imitate cement material, and thixotropy are important [41].

In light of the findings from this in-vitro study, laser metal sintering methods might be a more suitable alternative to conventional methods, especially in scenarios where the use of FDPs involves abutments with complex morphological features. However, this study did not consider the effects of veneer material, different framework materials, various cement types, and cementation techniques. Future studies should therefore take these factors and the dynamic oral conditions into account.

## Conclusions

Within the limitations of this in-vitro study, abutment type played a significant role in discrepancy values in the Cast method compared to that of the CAW method, in which the discrepancy values did not significantly affected from abutment type. Furthermore, the SLM and 3DP methods did not appear to be affected by abutment type with respect to marginal discrepancies. No effect of abutment type on occlusal discrepancies was observed in laser sinterization methods (DMLS and SLM). However, internal discrepancies in the SLM method might be influenced by abutment type.

Despite the significant differences between methods, the internal and occlusal discrepancies values might be expected similar when 3DP or Cast fabrication methods were preferred. Moreover, the effects of SLM, CAW, and DMLS fabrications methods on discrepancy values might be similarly. However, DMLS can be considered as a fabrication method for achieving optimal fit in terms of marginal, internal, and occlusal accuracy in three-unit FDPs, particularly in maxillary posterior regions.

## Data Availability

No datasets were generated or analysed during the current study.
